# Risk factors for poor physical and psychological outcomes after traumatic brachial plexus injury: a multicenter retrospective cohort study

**DOI:** 10.3389/fneur.2026.1880509

**Published:** 2026-09-03

**Authors:** Guang-Yao Li, Kang-Qi Xie, Jie Luo, Lang Cai, Er-Xi Xu, Ke Sha

**Affiliations:** 1 Department of Orthopedic Trauma and Hand Surgery, First Affiliated Hospital of Guangxi Medical University, Nanning, Guangxi, China; 2 Guangxi Key Laboratory for Preclinical and Translational Research on Bone and Joint Degenerative Diseases, Affiliated Hospital of Youjiang Medical University for Nationalities, Baise, Guangxi, China; 3 Orthopedics Department, The Second Nanning People's Hospital, Nanning, Guangxi, China

**Keywords:** brachial plexus injury, depression, neuropathic pain, risk factor, surgery

## Abstract

**Background:**

Traumatic brachial plexus injury (BPI) is associated with upper limb dysfunction, neuropathic pain (NP), and depressive symptoms, but factors linked to these outcomes remain insufficiently defined.

**Methods:**

This retrospective multicenter cohort study enrolled 327 surgical patients with BPI from 19 regional hospitals. Clinical data were collected from electronic medical records. Follow-up was performed through outpatient visits. Upper limb recovery was evaluated with the Medical Research Council (MRC) muscle strength scale, NP with the Visual Analog Scale (VAS), and depressive symptoms with the 9-item Patient Health Questionnaire (PHQ-9). Multivariable logistic regression was performed using SPSS. Model fit was assessed with the Hosmer-Lemeshow test and the area under the receiver operating characteristic curve (AUC).

**Results:**

Among 327 patients with unilateral BPI, 259 were male, and the mean age was 34.31 years. Traffic accidents (51.07%) were the main injury mechanism. Most injuries were closed (81.65%), and 81.04% patients had concomitant injuries, most frequently humeral fractures (36.70%). The most common injury pattern was C5-C6 (27.22%). Surgery was performed within 3 months in 195 patients. Over a mean follow-up of 45.78 months, 216 patients achieved muscle strength of M3 or higher. Independent risk factors for poor functional recovery included total BPI (OR = 4.62), surgical delay >6 months (OR = 4.41), and neurotization surgery (OR = 4.38). NP occurred in 189 patients; the main risk factors were surgical delay >6 months (OR = 6.76) and total BPI (OR = 6.22). Depressive symptoms were present in 192 patients; leading risk factors included motorcycle accident (OR = 6.18) and surgical delay >6 months (OR = 3.42). All models showed acceptable calibration and discrimination (AUC ≥ 0.76).

**Conclusion:**

Total BPI, root avulsion, and surgical delay beyond 6 months were significantly associated with adverse physical and psychological outcomes. Timely surgery and multidisciplinary care, including nerve repair, pain control, and psychological support, may improve recovery in high-risk patients with BPI.

## 1 Introduction

Brachial plexus injury (BPI) is one of the most devastating injuries affecting the upper limb, resulting in functional deficits and a high risk of permanent disability. In the early days, clinical outcomes of BPI were often poor, severely compromising both physical and mental health (1). Yeoman and Seddon (2) even proposed amputation of a nonfunctional limb rather than nerve repair surgery. Seddon (3) remained highly critical of BPI surgery, suggesting that treatment was appropriate mainly for upper trunk injuries and that the prognosis was discouraging. Encouragingly, since the mid-20th century, advances in microsurgery have driven the evolution of surgical strategies for BPI, leading to substantial improvements in functional recovery (4). Concurrently, neural regeneration and repair have greatly benefited from the development and application of artificial biomaterials (5). Nevertheless, the prognosis for severe BPI, particularly total root avulsion (RA), remains unsatisfactory (6).

On the other hand, the incidence of BPI has remained elevated, attributable to the increasing number of traffic accidents and advances in diagnostic techniques (7, 8). Midha (9) reported that among 4,538 consecutive polytrauma patients admitted to a Canadian trauma center from 1986 to 1994, 1.2% were diagnosed with BPI. Similarly, Suroto et al. (10) demonstrated a progressively rising annual incidence of BPI in Indonesia from 2003 to 2019. A higher incidence of BPI inevitably exposes more patients to the risk of neuropathic pain (NP) and depression. Evidence indicates that the prevalence of NP among BPI patients ranges from 67 to 95% (11), and such pain is chronic and treatment-resistant. Furthermore, depression affects 59.8% of patients with BPI, while anxiety and post-traumatic stress disorder are commonly observed (12). The adverse physical and psychological outcomes of BPI not only reduce quality of life but also pose major obstacles to clinical treatment and rehabilitation.

However, few studies have simultaneously explored the risk factors for limb functional recovery, NP, and depressive symptoms in surgical patients with BPI (13, 14). This highlights the lack of high-quality clinical evidence. Such evidence is essential for developing targeted strategies for BPI prevention, early diagnosis, and standardized treatment. Against this background, we conducted a multicenter study to analyze risk factors affecting the clinical outcomes of 327 patients with BPI.

## 2 Methods

### 2.1 Study population and design

This retrospective multicenter cohort study was approved by the Ethics Committee (approval no. 2016-E0023). A total of 337 patients with BPI underwent surgery at multiple medical centers, including 16 tertiary and 3 secondary hospitals, between January 2017 and December 2022. Inclusion criteria: (1) a definite history of trauma, with no upper time limit from injury to admission; (2) clinical symptoms and physical signs consistent with BPI; (3) supportive preoperative auxiliary examinations, including electromyography, ultrasound, CT, or MRI; (4) intraoperative findings confirming BPI. Exclusion criteria: (1) obstetric brachial plexus palsy; (2) non-traumatic brachial plexopathy, such as tumor-related, inflammatory, or radiation-induced plexopathy; (3) incomplete medical records; and (4) unavailable follow-up data.

### 2.2 Patient data

The following data were extracted from the electronic medical record system. General information included sex, age, occupation, educational level, and household registration. Clinical information included medical history, injury side, injury site, injury severity, and concomitant injuries. Surgical information included time from injury to surgery, surgical procedure, and intraoperative findings.

Injury patterns were classified by anatomical level and extent of involvement. Root avulsion was defined as a preganglionic injury in which one or more nerve roots were avulsed from the spinal cord. Total BPI was defined as involvement of C5 to T1. Partial BPI referred to injuries involving one or more, but not all, elements of the brachial plexus. Supraclavicular injuries involved the roots, trunks, or divisions, whereas infraclavicular injuries involved the cords or terminal branches. Cases with concomitant supra- and infraclavicular injuries were also recorded.

### 2.3 Follow-up survey

Patients were regularly followed up through outpatient visits, supplemented by telephone contacts to maintain long-term contact. Postoperative follow-up was scheduled at 3-month intervals in the first year, 6-month intervals from years 1 to 3, and annually thereafter, without upper time limit for patients with incomplete recovery. Upper limb function was assessed using the Medical Research Council (MRC) muscle strength scale, in which the degree of injury is indicated by the level of muscle paralysis, with muscle strength graded from 0 (no contraction) to 5 (maximum resistance movement). Successful recovery was defined as improvement in composite muscle strength for specified joint movement direction to grade M3 or higher (15). Once NP was confirmed, it was evaluated using the visual analog scale (VAS) and classified as mild (1–3), moderate (4–6), or severe (7–10) (16). Meanwhile, depression was assessed using the 9-item Patient Health Questionnaire (PHQ-9). Clinically relevant depressive symptoms were defined as a PHQ-9 score of 10 or higher (17). Nine patients were excluded (6 refused participation, and 3 lost contact), and one died from causes unrelated to the traumatic injury. A total of 327 patients were included in the final analysis.

### 2.4 Data analysis

Statistical analysis was performed using SPSS 22.0 (IBM Corp., Armonk, NY, United States). The normality of continuous variables was assessed via the Kolmogorov–Smirnov and Shapiro–Wilk tests. Normally distributed data were analyzed with the independent samples t-test, while non-normally distributed data were compared using the Mann–Whitney U test. Categorical variables were analyzed using the chi-square test or Fisher’s exact test.

Multivariable logistic regression analysis was conducted to identify risk factors for clinical outcomes. Results are presented as adjusted odds ratios (ORs) with 95% confidence intervals (CIs). Model calibration was evaluated using the Hosmer–Lemeshow test (H-L test, g = 10 subgroups, *p* > 0.05 indicating satisfactory model fit), and discriminative ability was assessed using the area under the receiver operating characteristic curve (AUC). McFadden’s pseudo-R^2^ was calculated to quantify goodness-of-fit. Multicollinearity was examined using variance inflation factor (VIF), with VIF < 5 indicating no collinearity, VIF 5–10 indicating moderate collinearity, and VIF > 10 indicating severe collinearity. Statistical significance was set at *p* < 0.05.

## 3 Results

### 3.1 Preoperative condition

**Figure 1:**
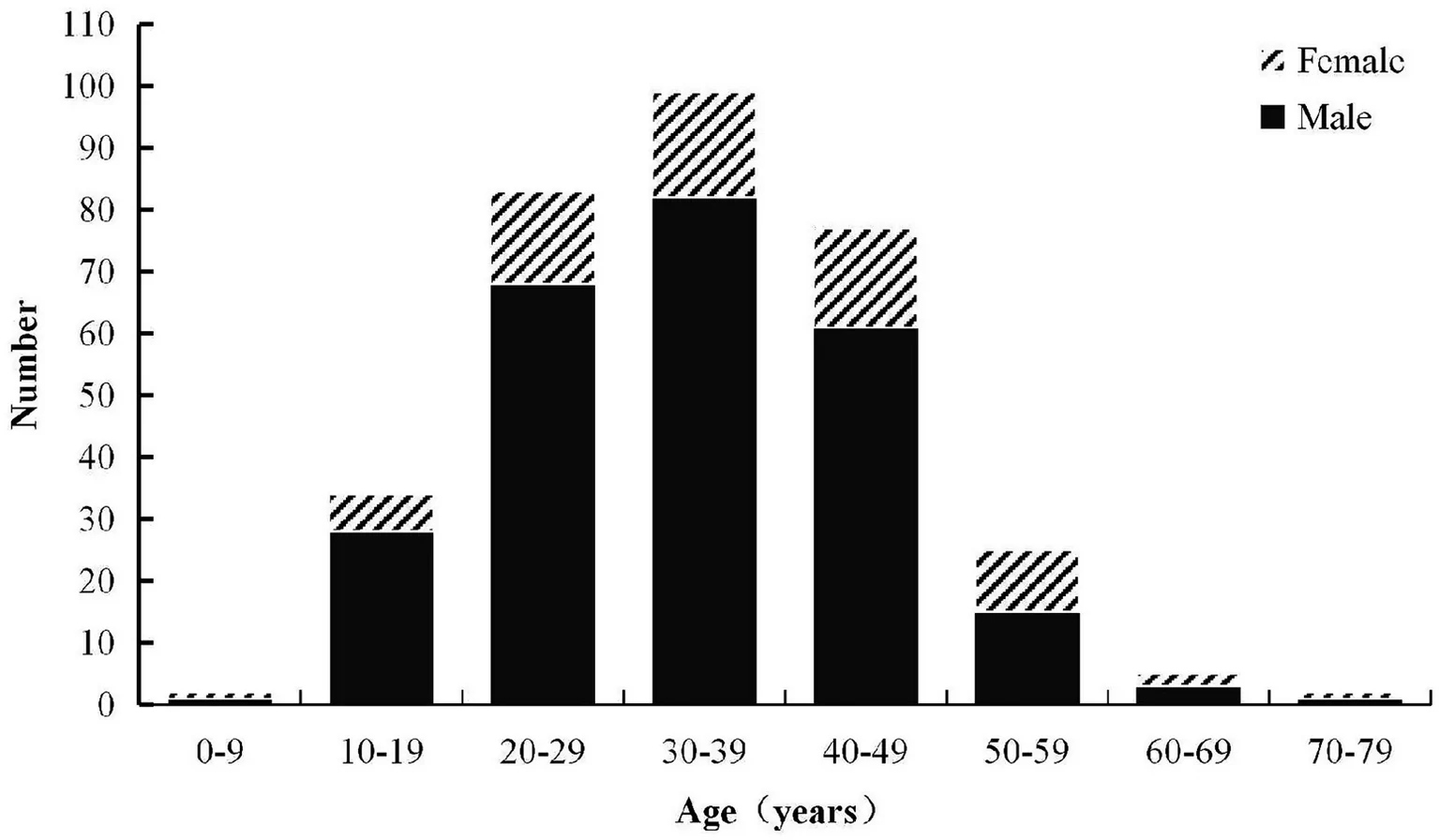
Age and sex distribution of patients with brachial plexus injury. Stacked bar chart illustrating the age and sex distribution of the study cohort. Age groups are presented in 10-year intervals from 0 to 79 years. Black bars represent male patients, and hatched bars represent female patients.

The majority of patients presented with closed injuries (*n* = 267, 81.65%). All BPI cases were unilateral, with 165 injuries on the left side (50.46%) and 162 on the right side (49.54%). This cohort showed marked male predominance (*n* = 259, 79.20%), with a male-to-female ratio of 3.81:1. The mean age was 34.31 years (range: 4–75 years), and the 30-39-year age group accounted for the largest proportion (30.28%, Figure 1). Traffic accidents were the leading cause of BPI (51.07%; Table 1), among which electric bike (e-bike) accidents accounted for 24.16% and motorcycle accidents for 19.27%.

**Table 1:** List and frequency of the injury mechanism of BPI.

Mechanism	No. of patients	Ratio (%)
Traffic accidents		
Motorcycle	63	19.27
Electric bike	79	24.16
Car	21	6.42
Other	4	1.22
Falls >2 m	53	16.21
Falls <2 m	17	5.20
Industrial injury	58	17.74
Sharp injury	14	4.28
Iatrogenic injury	12	3.67
Others	6	1.83

Of the 327 patients, 265 (81.04%) sustained concomitant injuries involving other anatomical regions (Table 2). Fractures were the predominant concomitant injury (*n* = 237), most frequently affecting the humerus (*n* = 120), ribs (*n* = 84), and scapula (*n* = 82; Table 3). Brain or spinal cord injury was recorded in 56 patients (17.13%) without severe symptoms. Additional neurological comorbidities included Horner’s sign (*n* = 57), trapezius paralysis (*n* = 32), hemidiaphragmatic palsy (*n* = 9), and winged scapula (*n* = 6).

**Table 2:** Concomitant injuries in patients with BPI.

Injuries	No. of patients	Percentage (%)
Fracture	237	72.48
Thoracic injury (excluding fractures)	61	18.65
Brain or spinal cord injury	56	17.13
Muscle injury or ischemic muscle spasm	23	7.03
Shoulder dislocation	23	7.03
Great vascular injury	21	6.42
Abdominal trauma	10	3.06

**Table 3:** Distribution of fractures associated with BPI.

fracture sites	No. of patients	Percentage (%)
Humerus	120	36.70
Ribs	84	25.69
Scapula	82	25.08
Clavicle	65	19.88
Bones of the forearm and hand	51	15.60
Spine	57	17.43
Bones of the lower limb	41	12.54
Skull	41	12.54
Pelvis	11	3.36

### 3.2 Surgery

A total of 195 patients underwent surgery within 3 months of injury, 70 underwent surgery at 3–6 months, and 62 underwent surgery after 6 months. Intraoperative findings revealed that the most frequent injury site was C5-C6 (27.22%). Other sites included C5-T1 (22.94%), cord/cord branch (20.80%), C5-C7 (19.27%). Concomitant supra- and infraclavicular injury (5.20%), C8-T1 (2.75%), and C7-T1 (1.83%). Among the 175 patients with nerve root, trunk, cord, or branch injuries, 41 presented with total RA, most frequently involving C5-C7 roots (Table 4). Neurolysis alone was the most common procedure (*n* = 152), followed by neurotization alone (*n* = 101; Table 5).

**Table 4:** Intraoperative findings in patients with BPI.

Injury sites	No. of patients			
	Avulsion	Rupture	Scar/neuroma in continuity	Scar/atrophy in continuity
Roots				
C5	75	11	85	16
C6	74	10	82	14
C7	69	4	48	1
C8	51	1	24	0
T1	46	0	16	0
Trunks				
Upper		20	63	5
Middle		5	38	3
Lower		1	49	0
Cord or cord to branch (infraclavicular)		18	53	14

**Table 5:** Initial surgical procedures performed in 327 patients.

Procedure	No. of patients
Neurolysis only	152
Neurotization only	101
Direct brachial plexus reconstruction with neurotization	42
Direct brachial plexus reconstruction	
End-to-end suture	10
Nerve grafting	15
Tendon transfer	4
Free functioning gracilis	3

### 3.3 Clinical outcomes and follow-up

The mean follow-up period was 45.78 ± 18.47 months. Among the 327 patients, 216 (66.06%) regained muscle strength of grade M3 or higher. Multivariable regression analysis identified seven independent risk factors associated with poor muscle strength recovery. The three major risk factors were total BPI (OR = 4.62), time from injury to surgery >6 months (OR = 4.41), and neurotization with or without reconstructive surgery (OR = 4.38, Table 6).

**Table 6:** Univariate and multivariate regression analyses of factors associated with limb functional outcomes in BPI patients.

Characteristic	Univariable					Multivariable				
	*N*	Event N	OR	95% CI	*p*-value^a^	*N*	Event N	OR	95% CI	*p*-value^a^
Age (years)	327	111	1.03	1.01, 1.05	0.003**	327	111	1.03	1.00, 1.05	0.022*
Sex										
Male	259	95	—	—		259	95	—	—	
Female	68	16	0.53	0.29, 0.98	0.044*	68	16	0.42	0.19, 0.91	0.029*
Injury mechanism										
Others	49	10	—	—		49	10	—	—	
Motorcycle	63	33	4.29	1.83, 10.06	<0.001***	63	33	3.52	1.21, 10.20	0.021*
Other traffic accident	104	27	1.37	0.60, 3.11	0.455	104	27	2.24	0.79, 6.33	0.129
Falls >2 m	53	25	3.48	1.45, 8.39	0.005**	53	25	2.92	1.00, 8.51	0.050*
Industrial injury	58	16	1.49	0.60, 3.66	0.390	58	16	0.96	0.32, 2.84	0.936
Injury type										
Open injury	60	24	—	—		60	24	—	—	
Closed injury	267	87	0.73	0.41, 1.29	0.274	267	87	0.76	0.36, 1.64	0.492
Brachial plexus injury										
Cord/Branch injury	68	12	—	—		68	12	—	—	
Partial root/trunk injury (partial BPI)	167	52	2.11	1.04, 4.27	0.038*	167	52	2.32	0.94, 5.73	0.068
Total root/trunk injury (total BPI)	75	38	4.79	2.22, 10.36	<0.001***	75	38	4.62	1.72, 12.43	0.002**
Concomitant supra- and infraclavicular injury	17	9	5.25	1.68, 16.39	0.004**	17	9	3.10	0.81, 11.87	0.098
Root avulsion										
No	232	61	—	—		232	61	—	—	
Yes	95	50	3.11	1.89, 5.12	<0.001***	95	50	3.19	1.56, 6.52	0.002**
Concomitant injury										
No	62	9	—	—		62	9	—	—	
Yes	265	102	3.69	1.74, 7.79	<0.001***	265	102	1.89	0.78, 4.54	0.157
Surgical procedure										
Neurolysis only	152	31	—	—		152	31	—	—	
Direct brachial plexus reconstruction	25	9	2.20	0.89, 5.44	0.089	25	9	2.80	0.92, 8.53	0.070
Neurotization with or without reconstruction	143	69	3.64	2.18, 6.08	<0.001***	143	69	4.38	2.14, 8.93	<0.001***
Functional reconstruction surgery	7	2	1.56	0.29, 8.43	0.605	7	2	2.84	0.40, 19.93	0.295
Time from injury to surgery										
0–3 M	195	42	—	—		195	42	—	—	
3–6 M	70	35	3.64	2.04, 6.50	<0.001***	70	35	3.23	1.58, 6.60	0.001**
> 6 M	62	34	4.42	2.41, 8.11	<0.001***	62	34	4.41	2.08, 9.33	<0.001***

a *p < 0.05; **p < 0.01; ***p < 0.001.

**Figure 2:**
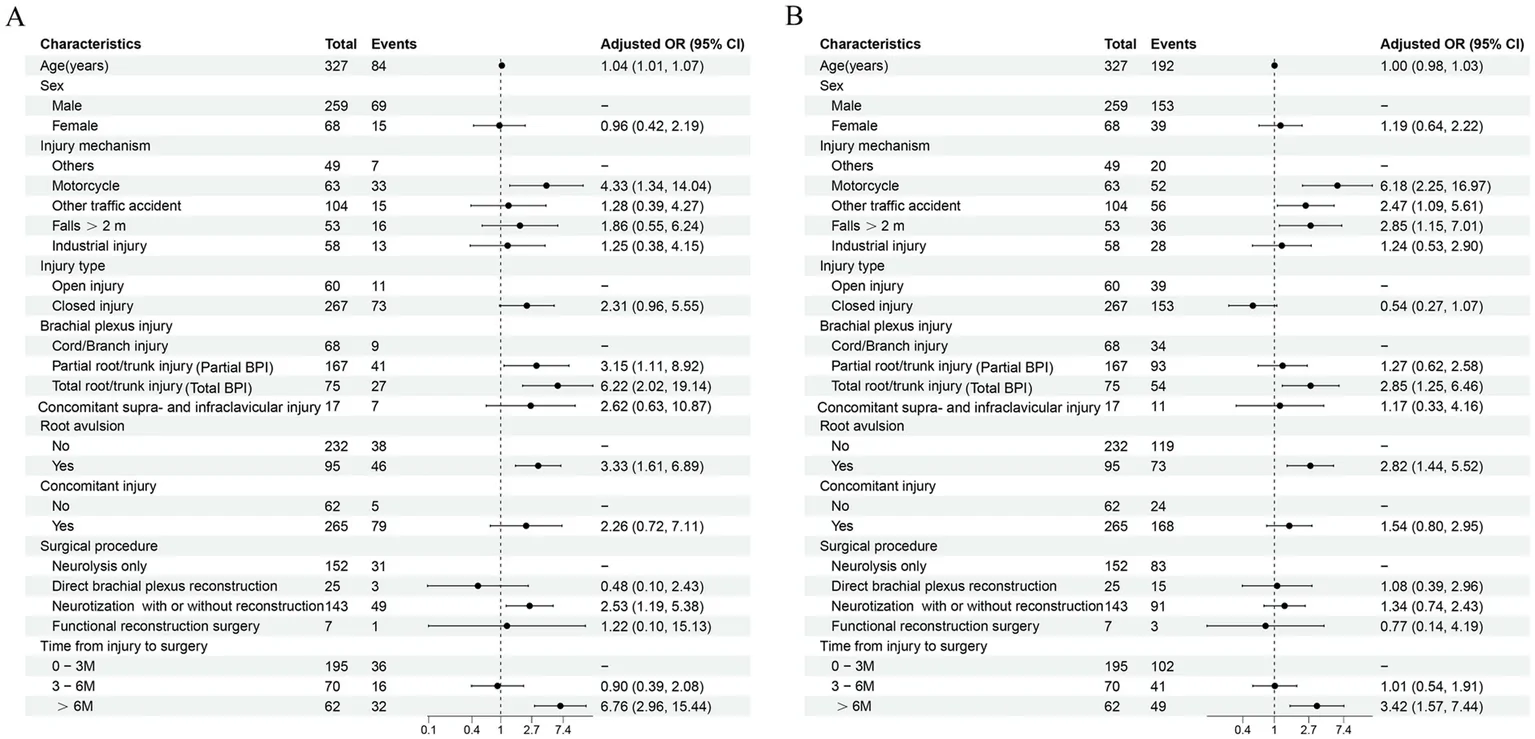
Multivariable logistic regression analyses of independent risk factors for neuropathic pain and depressive symptoms following BPI. **(A)** Multivariable logistic regression for moderate-to-severe neuropathic pain compared with no or mild pain. **(B)** Multivariable logistic regression for depressive symptoms compared with no depressive symptoms.

NP was occurred in 189 patients (57.80%). Among these patients, 105 (55.56%) had mild pain, 77 (40.74%) had moderate pain, and 7 (3.70%) had severe pain. Multivariable regression analysis identified six independent risk factors for moderate-to-severe NP. The three primary risk factors were time from injury to surgery >6 months (OR = 6.76), total BPI (OR = 6.22), and motorcycle accident (OR = 4.33), as shown in Figure 2A.

Depressive symptoms were observed in 192 patients (58.72%) during follow-up. Multivariable regression analysis identified six independent risk factors for depressive symptoms. The leading risk factors were motorcycle accident (OR = 6.18), time from injury to surgery >6 months (OR = 3.42), total BPI (OR = 2.85), and RA (OR = 2.82), as shown in Figure 2B.

All three models achieved good calibration with H-L test *p* > 0.05 and acceptable discriminative ability (AUC ≥ 0.76). The NP model exhibited the best performance (McFadden’s pseudo-R^2^ = 0.301, AUC = 0.854), whereas the depression model showed the lowest predictive performance (pseudo-R^2^ = 0.159, AUC = 0.760). No significant multicollinearity was observed across variables; all VIF values were below 10, with a maximum VIF of 6.73 (Table 7).

**Table 7:** Goodness-of-fit and multicollinearity diagnostics for the three binary logistic regression models.

Parameter	Model 1 Functional outcome	Model 2 Neuropathic pain	Model 3 Depression
Goodness-of-fit			
Sample size (*n*)	327	327	327
Event/non-event	111/216	84/243	192/135
McFadden pseudo-R^2^	0.282	0.301	0.159
AUC	0.837	0.854	0.760
Hosmer–Lemeshow χ^2^ (df = 8)	9.31	4.83	4.59
*p* value (H–L test)	0.317	0.776	0.800
Multicollinearity (VIF)			
Maximum VIF	6.73 (Age)		
Variables with VIF 5–10	Age (6.73), concomitant injury (5.65), injury type (5.24)		
Variables with VIF > 10	None		
Overall VIF range	1.15–6.73		

## 4 Discussion

### 4.1 Preoperative condition

In this study, BPI predominantly affected middle-aged males, with traffic accidents being the most common mechanism, consistent with previous reports (18, 19). However, there was a marked age-related difference in sex distribution. This phenomenon could be explained by occupational and behavioral patterns in this region: younger males are more likely to ride motorcycles or engage in heavy manual labor, increasing their exposure to high-energy trauma. In contrast, females more commonly use e-bikes, which are associated with lower-energy mechanisms (20). Additionally, older female patients are prone to BPI from low-energy falls, which is frequently associated with shoulder dislocation or upper extremity fractures (21, 22). In the past, such injuries were frequently missed due to limited diagnostic capabilities and technology. However, advances in clinical expertise and imaging modalities now enable early detection, which has significantly improved diagnosis rates, particularly among elderly patients. In addition, BPI caused by e-bike accidents were more frequent than those by motorcycle accident, indicating a notable shift in vehicle usage patterns.

The mechanism of injury directly affects the type of BPI, resulting in diverse clinical manifestations. Faglioni et al. (18) reported that total BPI were predominantly associated with firearm trauma (46.1%) in Brazil. Jain et al. (23) reported that two-wheeler accidents were responsible for 85.2% of BPI in India, with total BPI accounting for 48.4% of all cases. In contrast, this study revealed a markedly lower proportion of total BPI (22.94%), with higher rates of partial BPI (51.07%) and cord/branch injuries (20.80%). This characteristic is largely attributable to the lower-energy mechanism as the primary cause of BPI in our cohort.

Fractures were the most common concomitant injuries with BPI, particularly involving the long bones of the upper extremity and the shoulder girdle. These findings are consistent with previous reports (18, 24). Notably, the lower incidence of vascular injuries in this study compared with Western studies (24, 25), could be explained by differences in injury mechanism, as Western cohorts predominantly sustain higher-energy trauma. Additionally, supraclavicular injuries are more frequently associated with concomitant injuries than infraclavicular injuries, highlighting the importance of comprehensive evaluation to avoid missed diagnoses.

### 4.2 Surgery

Surgery is required for patients who present with evidence of nerve rupture or avulsion injury in the early stage, or for those whose affected limb muscle strength remains below grade M3 after 3 months of standardized non-surgical treatment. Intraoperative nerve action potentials (NAPs) were assessed after exposure of the involved BP elements. A bipolar stimulating electrode was placed proximal to the lesion, and the recording electrode was placed distal to the lesion. The presence of a reproducible propagated potential across the lesion was defined as a positive NAP. Neurolysis is recommended when intraoperative exploration confirms nerve with good continuity and a positive NAP (26). For traumatic neuromas with loss of conduction, tension-free neurorrhaphy following neuroma resection should be performed. Nerve grafting is recommended in the cases where direct coaptation cannot be achieved, as functional recovery deteriorates significantly when graft length exceeding 10 cm (27). Donor nerves for neurotization include both intraplexal and extraplexal sources. Intraplexal donors include the accessory nerve, ulnar or median fascicles, and the extensor carpi radialis brevis branch. Extraplexal donors include the intercostal nerve, phrenic nerve, and contralateral C7 nerve. Beyond 12–18 months post-injury, functional reconstruction is preferred over nerve repair, because distal degeneration, joint stiffness, and muscle fibrosis reduce the value of nerve repair (28).

### 4.3 Clinical outcomes

The clinical outcomes of this study are similar to those reported in previous studies (16, 24). Patients with infraclavicular injury achieved the best recovery (82.35%), followed by those with partial BPI (68.86%). The success rates for neurolysis alone, nerve suture, and nerve transfer were 79.61, 64.0, and 51.75%, respectively. These findings indicate that injury severity and lesion location are key determinants of functional recovery. Notably, no patient in this cohort had severe brain or spinal cord injury, so its potential effect on the accuracy of the results was limited. To facilitate the analysis of overall adverse outcomes after BPI, patients with concomitant injuries were grouped into a single category.

Multivariable logistic regression identified older age, male sex, motorcycle accident, total BPI, delayed surgery, RA, and neurotization surgery as independent risk factors for poor functional recovery, consistent with other studies (26, 29). Motorcycle accidents, fall from height, open injuries or the presence of concomitant injuries imply higher-energy trauma, which usually resulted in severe BPI. Additionally, older age and greater injury severity generally lead to worse recovery. The time from injury to surgery was confirmed as a strong predictor of functional outcome, with an optimal surgical window of within 6 months, as validated by Solla et al. (30). Delayed surgery exacerbates neurodegeneration, muscle fibrosis, and irreversible spinal cord remodeling, collectively contributing to poor limb function (31).

Interestingly, the significant association between neurotization surgery and poor functional recovery (OR = 4.38) appears contradictory to its moderate success rate (51.75%). This discrepancy likely reflects surgical indication rather than an inherent drawback of the procedure itself. Specifically, more severe injuries—particularly total RA—often compel surgeons to choose nerve transfer, and these patients are more prone to poor recovery. Therefore, clinicians should not avoid nerve transfer based on these findings; for severe BPI, nerve transfer may represent the only means of achieving meaningful functional outcomes. In addition, female sex emerged as a protective factor for functional recovery (OR = 0.42). This finding may be attributable to the fact that males are more frequently exposed to high-energy trauma-related activities, leading to intersex differences in the severity distribution of BPI.

Among 327 patients, 189 (57.80%) experienced NP, and moderate-to-severe pain often occurred in those with total BPI. Multivariable logistic regression identified older age, motorcycle accident, total BPI, RA, neurotization surgery, and surgical delay beyond six months as independent risk factors for NP. This finding is consistent with the cross-sectional study by Zhou et al. (32), who reported that more severe BPI was associated with a higher risk of NP. Motorcycle accidents are common causes of total BPI or RA. Total BPI typically leads to peripheral nerve degeneration and pathological neuroma formation, whereas RA additionally triggers ectopic discharge and central sensitization following the dorsal root entry zone injury. These mechanisms may make NP difficult to manage (33–35). Open injuries typically cause severe BPI, but they are mostly postganglionic. In contrast, closed injuries often result in preganglionic RA, which carries a higher risk of NP. Notably, Guo et al. (36) identified older age, in addition to high-energy mechanisms, as a risk factor for NP, aligning with the results of this study. This elevated risk may be attributed to age-related neurodegeneration, impaired analgesic function, diminished tissue repair capacity, and underlying comorbidities that facilitate pain sensitization after BPI.

Timely surgery could block the progression of peripheral degeneration and central sensitization to some degree, thereby significantly reducing the risk of NP (37). Once the injury exceeds 6 months, prolonged denervation induces structural remodeling of the spinal dorsal horn, persistent sensitization of dorsal root ganglion neurons, and cross-regional reorganization of the sensory cortex, leading to intractable phantom limb pain and dysesthesia that are difficult to reverse (11, 38, 39)—a conception consistent with our finding. However, nerve transfer was found to be a risk factor for NP (OR = 2.53), may reflect both patient selection bias, as well as the iatrogenic donor nerve injury associated with the procedure (34). Moreover, although male patients sustained more severe injuries, sex was not a risk factor for NP. This may be related to greater pain sensitivity in females, as well as sex differences in pain treatment responses (40). Currently, neither pharmacologic nor surgical interventions provide sustained relief for NP (41). Therefore, early detection and comprehensive treatment in high-risk patients represent the only effective strategy.

On the other hand, 192 patients (58.72%) exhibited depressive symptoms. This proportion was slightly higher than the 45.9% prevalence of depression reported by Gaston et al. (42). This discrepancy may be attributable to differences in assessment tools and to the limited availability of mental health support resources in this region. Meanwhile, several studies have demonstrated that adverse psychological outcomes after BPI are affected by multifactorial elements, including individual factors such as negative coping strategies and persistent pain, as well as environmental factors like social dysfunction and upper limb functional impairment (43, 44). All of these factors deserve more attention.

Multivariable logistic regression revealed that traffic accidents, fall from height, total BPI, RA, and surgical delay beyond six months were independent risk factors for depression. More severe BPI often result in worse upper limb dysfunction and more intense NP, which jointly contribute to a higher risk of psychological disorders (32, 42). Notably, surgical delay beyond six months increased the risk of depression approximately threefold (OR = 3.42), a finding of considerable clinical significance. Martin et al. (37) reported that timely operation confers protective psychological benefits, suggesting that expedited surgical referral is not only a functional recovery strategy but also an effective psychological intervention in itself. In contrast to other models, traffic accidents and falls (>2 m) significantly increased risk of depression. This association may be attributed to severe psychological trauma triggered by intense fear and a sense of loss of control experienced at the time of injury.

Notably, neurotization surgery was not identified as an independent risk factor for depressive symptoms. The discriminative ability of the depression model was lower than that of the functional and NP models. This suggests that psychological outcomes are influenced by factors beyond injury characteristics alone (45). The impact of social factors on psychological prognosis was independent of injury severity, highlighting an issue that warrants proactive intervention. Furthermore, all three models showed good calibration, acceptable discriminatory power, and no obvious multicollinearity, supporting their use for identifying risk factors in surgical patients with BPI.

### 4.4 Limitations

This study has several limitations. First, its retrospective multicenter design has inherent limitations, including possible variation in treatment protocols and follow-up procedures across centers, although all procedures were performed under the supervision of our team. Second, dominant-limb involvement was not collected for further analysis. This variable may be important because injury to the dominant upper limb can have a greater effect on function, daily activities, and quality of life. Third, although VAS is useful for grading pain intensity, it is not specific for neuropathic pain. Fourth, PHQ-9 records depressive symptoms only during the previous two weeks, which may not reflect the long-term psychological burden after BPI. Finally, preoperative psychological status was not systematically assessed, which prevented comparison of psychological status before and after surgery. Future prospective studies should minimize these inherent limitations by collecting more comprehensive data, using more precise assessment tools before and after surgery, and analyzing both short-term and long-term outcomes to better identify prognostic factors in patients with BPI.

## 5 Conclusion

This multicenter retrospective study shows that total BPI, root avulsion, and surgical delay beyond 6 months are associated with poor functional recovery, NP, and depressive symptoms in surgical patients. Different injury mechanisms had different associations with pain and psychological outcomes, possibly reflecting both injury severity and acute psychological trauma of BPI. The association between neurotization surgery and unfavorable functional outcome likely reflects surgical indication bias rather than a procedural limitation. The lower discriminative performance of the depression model highlights the contribution of social and psychosocial factors beyond injury characteristics alone. These findings are consistent with the clinical value of early diagnosis and multidisciplinary care that integrates timely surgery, pain management, and psychological support, particularly for patients with high-energy trauma, total BPI, or RA.

## Data Availability

The raw data supporting the conclusions of this article will be made available by the authors, without undue reservation.
